# The role of inhibitory immune checkpoint receptors in the pathogenesis of Alzheimer’s disease

**DOI:** 10.1007/s00109-024-02504-x

**Published:** 2024-11-27

**Authors:** Antero Salminen

**Affiliations:** Department of Neurology, Institute of Clinical Medicine, University of Eastern Finland, P.O. Box 1627, FI-70211 Kuopio, Finland

**Keywords:** Ageing, Alzheimer’s disease, Immune tolerance, Immunosenescence, Immunosuppression, Microglia

## Abstract

**Abstract:**

There is mounting evidence that microglial cells have a key role in the pathogenesis of Alzheimer’s disease (AD). In AD pathology, microglial cells not only are unable to remove β-amyloid (Aβ) plaques and invading pathogens but also are involved in synaptic pruning, chronic neuroinflammation, and neuronal degeneration. Microglial cells possess many different inhibitory immune checkpoint receptors, such as PD-1, LILRB2-4, Siglecs, and SIRPα receptors, which can be targeted by diverse cell membrane-bound and soluble ligand proteins to suppress the functions of microglia. Interestingly, in the brains of AD patients there are elevated levels of many of the inhibitory ligands acting via these inhibitory checkpoint receptors. For instance, Aβ oligomers, ApoE4, and fibronectin are able to stimulate the LILRB2-4 receptors. Increased deposition of sialoglycans, e.g., gangliosides, inhibits microglial function via Siglec receptors. AD pathology augments the accumulation of senescent cells, which are known to possess a high level of PD-L1 proteins, and thus, they can evade immune surveillance. A decrease in the expression of SIRPα receptor in microglia and its ligand CD47 in neurons enhances the phagocytic pruning of synapses in AD brains. Moreover, cerebral neurons contain inhibitory checkpoint receptors which can inhibit axonal growth, reduce synaptic plasticity, and impair learning and memory. It seems that inappropriate inhibitory immune checkpoint signaling impairs the functions of microglia and neurons thus promoting AD pathogenesis.

**Key messages:**

- Microglial cells have a major role in the pathogenesis of AD.
- A decline in immune activity of microglia promotes AD pathology.
- Microglial cells and neurons contain diverse inhibitory immune checkpoint receptors.
- The level of ligands for inhibitory checkpoint receptors is increased in AD pathology.
- Impaired signaling of inhibitory immune checkpoint receptors promotes AD pathology.

## Introduction

For decades, it has been known that an accumulation of β-amyloid (Aβ) plaques within the brain and neurofibrillary tangles in neurons are the classical hallmarks of Alzheimer’s disease (AD). The synthesis of Aβ precursor protein (APP), its processing into Aβ peptides, and their deposition into amyloid plaques have been the major focus of AD research. The amyloid cascade hypothesis as an etiology of AD was presented over 30 years ago [1]. During recent years, many immunotherapeutic trials have attempted to remove Aβ plaques from the brains of AD patients [2]. Unfortunately, the results have been modest although the aducanumab treatment has been approved for clinical use in AD therapy [3]. Currently, there are diverse approaches to discover therapies which could prevent AD pathology and alleviate cognitive impairments [4].

Many genetic studies have indicated that the immune system has a crucial role in the pathogenesis of AD [5, 6]. In particular, the genome-wide studies have highlighted the role of microglia in the pathogenesis of AD since several risk genes for AD pathology are expressed in microglial cells [7–9]. Immune surveillance and phagocytosis of damaged structures and invaded pathogens are crucial functions of microglia in the brain [10, 11]. Given that there is deposition of Aβ plaques within AD brain, this indicates that microglia are either hyporesponsive to undertake immunosurveillance and induce phagocytosis or alternatively Aβ plaques are masked by “self” markers. The inhibitory immune checkpoint pathway is the master immune mechanism in immune surveillance and thus in the maintenance of tissue homeostasis [12, 13]. In the brain, microglial cells contain diverse inhibitory checkpoint receptors and interestingly, AD pathology displays an elevated amount of their ligands which are able to suppress the immune surveillance capacity of microglia and thus prevent the clearance of Aβ plaques (see below). Here, I will examine the function of microglial inhibitory checkpoint signaling in AD pathology, and it seems that the inhibitory immune checkpoints have an important role in AD pathogenesis.

## Hyporesponsive microglia in AD pathology

Microglia are the main determinants of immune functions in AD pathology [11, 14, 15]. Microglia are versatile immune cells which in pathological conditions can display both pro-inflammatory and anti-inflammatory properties. Microglia are extremely plastic cells not only in their morphological characteristics but also in their functional responses, e.g., in immune surveillance, antigen presentation, phagocytosis, and synapse pruning, as well as in promoting inflammation and its resolution. Microglia act in close cooperation with astrocytes, also called astroglia, nerve cells, tissue-resident innate lymphoid cells, and the immune cells which gain access to the brain in pathological conditions. The interactions with other cells not only are based on the secretion of cytokines, chemokines, and proteolytic enzymes but can also occur through the cell–cell contacts mediated via their activating and inhibitory immune checkpoint receptors. Furthermore, microglial cells can also act as immunomodulators by secreting extracellular vesicles [16]. It is not surprising that the single-cell RNA sequencing techniques have revealed that there is an extensive heterogeneity in the microglial subpopulations detected in AD [17–20]. It seems that microglial transcriptomes can take multiple trajectories during the progression of AD. For instance, Prater et al. [20] identified ten different microglial clusters with only one subcluster being specific for AD patients. Some other investigators have also reported the presence of AD-specific microglia clusters [17, 18]. Keren-Shaul et al. [17] identified a novel disease-associated microglial (DAM) population in AD which was detected in both humans and transgenic AD mice. This phenotype restricted the development of AD by activating triggering receptor expressed on myeloid cell 2 (TREM2)-dependent signaling in conjunction with the suppression of some inhibitory immune pathways. However, single-cell studies on microglial populations in AD have not been repeatedly able to reveal convincingly any specific AD-related microglial phenotype.

Currently, the role of microglia in the generation and clearance of Aβ plaques in AD pathology is somewhat unclear although it is known that microglial cells can avidly phagocytoze Aβ deposits. There are many investigations indicating that microglial cells have a crucial role in the formation and surveillance of Aβ plaques in AD brains [21, 22]. Moreover, it has been claimed that peripheral monocytes and macrophages could target Aβ plaques and even participate in their removal from the AD brain [23–25]. Nonetheless, there is abundant evidence that the chronic neuroinflammatory state present in AD brains promotes a state of immune hyporesponsiveness of microglial cells towards Aβ deposition and impairs their ability to phagocytize the accumulating Aβ plaques [26–29]. For instance, Krabbe et al. [28] demonstrated that the phagocytic activity of cortical microglia was clearly impaired in transgenic APP/presenilin 1 (PS1) and APP23 mice. They also reported that anti-Aβ exposure restored phagocytic activity of microglial cells and reduced the load of Aβ plaques in APP/PS1 mice. Interestingly, the overexpression of pro-inflammatory cytokines, e.g., IL-1β and TNF-α, significantly decreased Aβ deposition in the brains of transgenic AD mice [30, 31]. It seems that the AD-related immunosuppression of microglia can be mitigated by increasing inflammatory insults and thus enhancing their phagocytic properties. On the other hand, there are reports that an overexpression of immunosuppressive IL-4, IL-10, and TGF-β cytokines in the brains of transgenic AD mice clearly stimulated the accumulation of Aβ plaques [32–34]. These results imply that the immunosuppressive state of microglia promotes the deposition of Aβ plaques within AD brains.

## Inhibitory immune checkpoint receptors

The lymphoid and myeloid cells of the immune system have both stimulatory and inhibitory immune checkpoint receptors which drive immune surveillance. This balance is important when these cells confront invading pathogens as well as aberrant host cells, such as damaged, senescent, and malignant cells [12, 13, 35]. Moreover, the checkpoint immunoreceptors have a crucial role in the maintenance of tissue self-tolerance which prevents the host’s tissues from being attacked by immune cells. The inhibitory checkpoint receptors are ligand-activated immune receptors which contain extracellular recognition domains, such as immunoglobulin-like V domains, and a cytoplasmic tail with both immunoreceptor tyrosine-based inhibitory motifs (ITIM) and tyrosine-based switch motifs (ITSM) [13, 36, 37]. Accordingly, the stimulatory immune receptors contain the recognition domain but the cytoplasmic tail represents an immunoreceptor tyrosine-based activation motif (ITAM). When a ligand binds to the inhibitory checkpoint receptor, this stimulates the phosphorylation of their intracellular ITIM and ITSM motifs, thus opening the docking sites for the Src-homology protein phosphatases, such as SHP-1, SHP-2, and SHIP [13, 36]. Consequently, these three activated phosphatases (SHP-1, SHP-2, and SHIP) are able to inhibit a myriad of signaling cascades and thus impair many functions of immune cells [38–40]. For instance, the activation of SHP-1 phosphatase can suppress the signaling through the T-cell receptor (TCR) and the B-cell receptor (BCR) [13, 38]. It is known that SHP-1 inhibits the activation of PI-3 K and ZAP70 signaling and thus suppresses the functions mediated through the TCR axis. Accordingly, SHIP1 can also attenuate several immune functions by inhibiting the MAPK, PI-3 K and NF-κB signaling pathways [41]. In particular, PI-3 K signaling not only drives energy and protein metabolic pathways but also regulates many responses of innate and adaptive immunity [42]. Moreover, some immunosuppressive cytokines, e.g., IL-10 and TGF-β, can activate SHP-1 phosphatase [43, 44] which means that the signaling pathways of inhibitory checkpoint receptors and immunosuppressive cytokines secreted by myeloid-derived suppressor cells (MDSC), regulatory T cells (Treg), and M2 macrophages merge in the activation of immunosuppressive phosphatases, i.e., SHP-1, SHP-2, and SHIP.

**Fig. 1:**
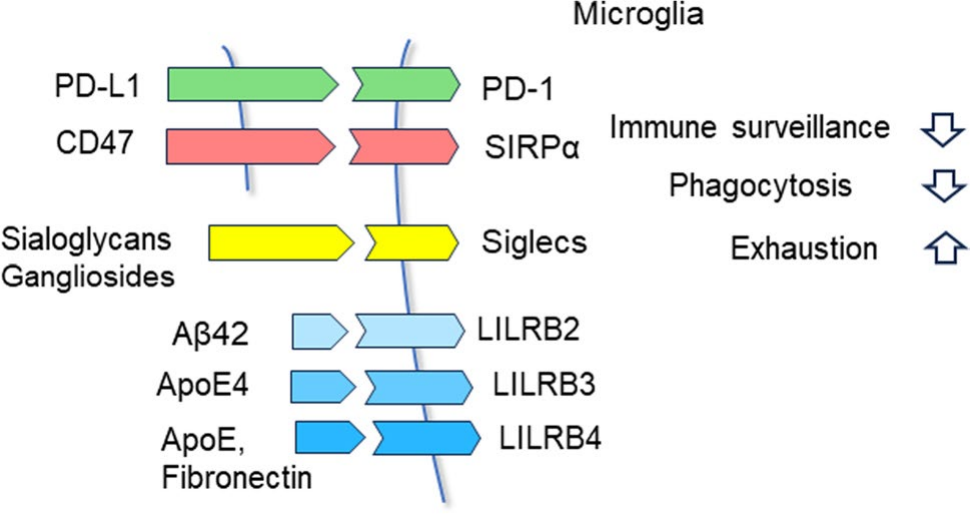
A schematic figure depicting the inhibitory immune checkpoint receptors in microglial cells and their ligand proteins which are known to be present in the brains of AD patients. Activation of microglial inhibitory checkpoint receptors impairs their immune surveillance efficacy and phagocytic activity as well as can lead even the exhaustion of microglia. Abbreviations: Aβ42, amyloid-β(1–42); ApoE, apolipoprotein E; CD47, cluster of differentiation 47; LILRB, leukocyte immunoglobulin like receptor B; PD-1, programmed cell death protein-1; PD-L1, programmed death-ligand 1; Siglecs, sialic acid-binding immunoglobulin-type lectins; SIRPα, signal regulatory protein α

There are many membrane-bound and soluble ligand proteins which can act to inhibit the activities of the inhibitory checkpoint receptors of immune cells (see the next Section). These ligands are a part of an elaborate system aiming to maintain immune tolerance and deficiencies in that signaling system promote the development of autoimmune diseases. The major histocompatibility complex (MHC) system, also known as human leukocyte antigens (HLA) in humans, is the basis of self-tolerance and a defense against unrecognized antigens. However, many inhibitory checkpoint signaling pathways act as co-inhibitory pathways for the TCR and BCR systems, such as the programmed cell death protein-1 (PD-1)/programmed death-ligand 1 (PD-L1) axis (see below), which suppresses immune surveillance via activation of SHP-1, SHP-2, and SHIP phosphatases (Fig. 1). Loss of cytotoxicity of surveying CD8^+^ T and natural killer (NK) cells, leading even to exhaustion, apoptosis, and anergy of T cells, is a typical immune response caused by the activation of certain inhibitory checkpoint receptors [13, 45, 46]. However, there are significant differences in the expression levels of inhibitory checkpoint receptors between different immune cells, e.g., in pathological conditions. Furthermore, the inactivation of effector immune cells by inhibitory checkpoint pathways can produce specific hypoactivity states or interestingly, the activation of the checkpoint receptors, e.g., the PD-1/PD-L1 signaling axis, can trigger the differentiation of CD4^+^ T cells into immunosuppressive Treg cells [47, 48]. Currently, the signaling mechanisms which promote the immunosuppressive differentiation stimulated by inhibitory checkpoint receptors need to be clarified although it is known that SHP-1, SHP-2, and SHIP are involved, and thus, these phosphatases are considered important targets in drug discovery projects for cancer and autoimmune disorders [49].

Genetic studies have revealed that several risk genes of AD are expressed in microglia and they are involved in microglial-evoked inflammation [7, 9]. Certain genes, such as CD33/Siglec-3, apolipoprotein E (ApoE), and INPP5D/SHIP1, are associated with the functions of inhibitory checkpoint receptors (see below). It was reported that a polymorphism in the *INPP5D* gene increased an individual’s risk of suffering AD pathology [50]. However, given that SHIP1 has a crucial role in the regulation of cellular differentiation and survival pathways [40], it is not known whether these pathways are associated with inhibitory checkpoint signaling. Next, I will review the properties and functions of those inhibitory checkpoint receptors and their ligands which are known to be expressed in both microglia and some neurons as well as to be involved in the pathogenesis of AD (Fig. 1). In addition to the PD-1/PD-L1 axis, there are several other receptor/ligand pathways, i.e., (i) Siglecs/sialoglycans; (ii) leukocyte immunoglobulin like receptor B (LILRB2-4) and their ligands Aβ, ApoE, and fibronectin; and (iii) signal regulatory protein α (SIRPα) and its ligand, the cluster of differentiation 47 (CD47) protein. Moreover, human and mouse microglial cells possess certain other inhibitory immune checkpoint receptors, e.g., lymphocyte-activation gene 3 (LAG3) and V-domain Ig suppressor of T cell activation (VISTA), but their role in AD pathogenesis still needs to be clarified.

## Inhibitory immune checkpoint receptors in microglia

### PD-1 receptor

The PD-1 receptor (CD279) is expressed in immune cells, especially in T and B cells, macrophages, and natural killer (NK) cells as well as in microglial cells [51–53]. Moreover, it is known that the functional PD-1 receptors are also widely expressed in neurons, especially in sensory neurons [54, 55]. The PD-1 protein is a cell-surface inhibitory checkpoint receptor which contains an extracellular IgV domain and a cytoplasmic protein sequence with one ITIM and one ITSM domain. It is known that there are two ligands which activate the PD-1 receptor, i.e., PD-L1 (CD274 or B7-H1) and PD-L2 (CD273 or B7-DC), which have been characterized as immune regulatory proteins [56]. The PD-L1 protein is expressed in some immune cells and in many stromal cells, e.g., in astrocytes, pericytes, and endothelial cells in the brain [57, Human Protein Atlas]. Interestingly, the PD-L1 receptor is also expressed in microglia which means that microglial cells are able to suppress the activity of immunosurveying immune cells in pathological states [58, 59]. For instance, Schachtele et al. [58] demonstrated that experimental murine cytomegalovirus-induced encephalitis stimulated the expression of PD-L1 in microglia in mouse brain. They revealed that microglia inhibited the cytotoxic activity of infiltrating CD8^+^ T cells via the PD-1/PD-L1 pathway. There is robust evidence that the activation of the PD-1 receptor in microglia via PD-L1 signaling can suppress neuroinflammation in many pathological conditions, such as ischemic stroke, traumatic brain injury, Alzheimer’s disease, and multiple sclerosis [52, 53, 60, 61]. The activation of the PD-1 receptor in microglia stimulates inhibitory signaling through SHP-1 and SHP-2 which subsequently inhibit the ERK, NF-κB, and STAT1 pathways and thus dampen the severity of inflammatory responses. Accordingly, it has been demonstrated in cell cultures and mouse brain that a deficiency of PD-1 signaling stimulated the polarization of microglia into the pro-inflammatory M1 phenotype [62].

### Siglec receptors

Siglec receptors are sialic acid-binding Ig-like lectins which specifically recognize different sialoglycan components on the glycocalyx of immune and non-immune cells [63]. The Siglec receptors undertake many functions not only in cell adhesion and phagocytosis but especially in the regulation of crucial immune functions [64–66]. Most of the human and mouse Siglec receptors contain the ITIM or ITIM-like binding domains for SHP-1 and SHP-2 phosphatases, i.e., they are inhibitory immune checkpoint receptors [63]. The CD33 (Siglec-3)-related inhibitory receptor family contains human Siglec-3 and Siglec-5 to Siglec-11 members. Instead, the Siglec-16 receptor does not contain the ITIM domain but associates with the DAP12 protein which houses the ITAM domain, thus Siglec-16 transduces activating signals [67]. There are many structural and functional differences between the human and mouse Siglec receptors. Several inhibitory Siglec receptors have been observed in human microglia although the Siglec-3, Siglec-8, and Siglec-11 receptors seem to be the major inhibitory checkpoint receptors [68–70]. Interestingly, the Siglec-3/CD33 and Siglec-11 are expressed exclusively in microglial cells in adult human brain [68, 71]. Wang et al. [68] demonstrated that the expression of Siglec-11 receptor evolved in the later evolutionary stages of the hominin brain, after the emergence of the genus *Homo*. It should be recalled that the appearance of Aβ plaques and neurofibrillary tangles are rarely encountered in non-human primates, e.g., in the brains of aged orangutans [72]. The activation of Siglec receptors in microglia, as many other inhibitory checkpoint receptors, inhibits pro-inflammatory responses and downregulates phagocytosis although concurrently it exerts certain neuroprotective properties [73, 74]. Disturbances in the function of Siglec/sialoglycan axis have been associated with many neurodegenerative processes, such as axon demyelination, synaptic loss, and Aβ aggregation in AD [74, 75].

There is robust evidence that sialylation is a crucial regulator of many innate immune responses in the central nervous system [66, 74, 76–78]. Gangliosides are a rich source of sialic acids in the brain since about 75% of the total sialic acid content is located in the gangliosides present in the mammalian brain [79]. For instance, tissue sialoglycans can control the immune tolerance of tissues, e.g., the cancer microenvironment houses an abundance of sialic acids which cancer cells exploit to mimic the “self” recognition patterns [76, 80]. The use of sialylated glycan microarrays has revealed the high specificity of different Siglec receptors for sialoglycan structures [66, 81]. There are reports detailing the structures of sialylated ligand molecules which target the Siglec receptors present in human microglia, i.e., Siglec-3, Siglec-8, and Siglec-11 [70, 82, 83]. For instance, human Siglec-3 recognizes the α2,3 and α2,6 sialic acids present on the terminal sugar backbone, whereas Siglec-11 binds to the α2,8 sialic linkage and the homomeric polysialic backbone [82–84]. Sialyltransferases and sialidases/neuraminidases can modify the structures of tissue sialoglycans, and thus, these enzymes can affect the immune properties of tissue sialoglycans. The changes in the sialylation of glycocalyx are able to activate the inhibitory Siglec checkpoints and thus they can (i) inhibit neuroinflammation, (ii) regulate phagocytosis, and (iii) control the cytotoxicity of CD8^+^ T and natural killer (NK) cells [64, 76].

### LILRB2-4 receptors

Human LILRB receptors are inhibitory immune checkpoint proteins which contain two to four cytoplasmic ITIM domains [85, 86]. Interestingly, there are also LILRA receptors which instead of inhibiting, promote the activation of certain immune cells [85]. The LILRB2-4 receptors are primarily expressed in many immune cells including microglial cells in human and mouse brain [85, 87–91]. Additionally, there is substantial evidence that LILRB receptors are expressed in neurons, especially the function of murine homolog of LILRB2; i.e., the paired immunoglobulin-like receptor B (PirB) has been studied in connection with the synaptic plasticity of neurons [87, 92, 93]. The human LILRB receptors, totally five different proteins, have homologous structural domains and many important functions which have been attributed to their cytoplasmic ITIM domains. In general, the activation of the LILRB2-4 receptors promotes immune tolerance which has beneficial effects in autoimmune diseases and transplantation biology although it drives many harmful outcomes in chronic infections and tumors [85, 86]. For instance, an increase in the expression of the LILRB4 receptor reduced the expression and secretion of cytokines and chemokines in several experimental models [86]. The activation of LILRB4 also suppressed the phagocytosis of opsonized bacteria in human THP-1 cells [94]. Moreover, it was demonstrated that LILRB4 signaling enhanced the differentiation and immunosuppressive activity of mouse MDSCs and consequently promoted metastasis of tumor cells [95]. There is abundant evidence indicating that activation of LILRB2-4 receptors in immune cells promoted immune tolerance of tissues by preventing pro-inflammatory responses and enhancing the immunosuppressive activities of MDSCs, Tregs, and M2 macrophages [85, 86, 95, 96].

Although the signaling pathways and functions of the LILRB2-4 receptors resemble each other in microglia, there are significant differences in the binding of specific ligands and also in the expression levels [85]. For instance, several histocompatibility antigens, e.g., HLA-G, have a high affinity for the LILRB2 receptor but not for the LILRB3 and LILRB4 receptors. Angiopoietin-like proteins 2 and 5 (ANGPTL2/5) are the ligands for the LILRB2 and LILRB3 receptors but not for the LILRB4 receptor. The CD166 (ALCAM) transmembrane glycoprotein, which is expressed in activated T cells, epithelial cells, and fibroblasts, is a specific ligand for the LILRB4 receptor [97]. With respect to AD pathology, there are some interesting ligands for the LILRB2-4 receptors; i.e., (i) Aβ1–42 (Aβ42) oligomers target LILRB2 [87, 98], and (ii) ApoE4 is a ligand for LILRB3 [86], whereas (iii) ApoE and fibronectin are ligands for the LILRB4 receptor [85, 99, 100].

### SIRPα receptor

The SIRPα protein is a cell surface-bound inhibitory checkpoint receptor which is mostly expressed in myeloid cells, such as macrophages and microglia [101, 102]. Moreover, the SIRPα protein is a multifunctional glycoprotein, also called the SH2 domain-containing protein tyrosine phosphatase substrate 1 (SHPS1), which is a synapse-associated membrane protein expressed in neurons [103, 104]. The SIRPα protein interacts with the CD47 protein, a widely expressed “don’t-eat-me” signal, which suppresses the SIRPα-induced phagocytosis via its cytoplasmic ITIM domain [101]. Cancer cells and senescent cells express an increased level of the CD47 protein to avoid phagocytic clearance by immune cells [101, 105, 106]. Although the SIRPα receptor is a major anti-phagocytic receptor, many other inhibitory checkpoint receptors possess anti-phagocytic properties, such as PD-1, LILRB1, and many Siglec receptors [107]. There are observations that microglial cells express the SIRPα protein in different experimental conditions [102, 108, 109]. In the brain, the microglial SIRPα protein acts in the pruning process, i.e., the elimination of synapses, during the developmental phase [110], and this has been observed also in experimental models of neurodegeneration [102]. Gaikwad et al. [111] reported that the human and mouse microglial cells also expressed the SIRPβ1 protein which was able to increase the phagocytosis of neural debris and Aβ deposits. Ding et al. [102] observed that a specific ablation of the microglial SIRPα protein decreased the synaptic density in mouse brain. They also revealed that a loss of SIRPα increased the phagocytosis of synaptic structures by the SIRPα deficient microglia. Ding et al. [102] also reported that a downregulation of the level of the CD47 protein facilitated the microglia-mediated synaptic clearance in mouse brain. Given that the expression of the CD47 protein is increased in many diseases, e.g., in atherosclerosis, Kojima et al. [112] demonstrated that the blocking antibody therapy to the CD47 protein restored the phagocytic activity of macrophages and reduced the severity of atherosclerosis in experimental mouse models. It seems that the SIRPα/CD47 axis is a potent inhibitor of phagocytosis, i.e., an increase either in the expression of microglial SIRPα or in the level of neuronal CD47 suppresses phagocytic activity.

### LAG3 and VISTA receptors

There are also certain inhibitory immune checkpoint receptors which are expressed in microglia but currently their role in AD pathology is unclear or there is not enough evidence for their involvement in AD pathology. The LAG3 (CD223) receptor is a type I transmembrane protein possessing four extracellular Ig-like domains and three evolutionarily conserved signaling motifs which mediate the inhibitory signaling of the LAG3 receptor [113, 114]. The LAG3 protein is expressed at a high level in activated T cells where it acts as an immune co-receptor inhibiting the signaling via the TCR pathway, and thus, it suppresses the activation of T cells. In the brain, the LAG3 receptor is mostly expressed in microglial cells [115, 116]. Morisaki et al. [115] revealed that the expression of LAG3 is under the control of IFN-γ/STAT1 signaling and IFN-γ exposure robustly increased the expression of the LAG3 protein in murine microglial cells. They also reported that two metalloproteinases, ADAM10 and ADAM17, cleaved the soluble LAG3 peptide from the membrane-bound LAG3 receptor. These results imply that a strong expression of the LAG3 protein induced by the IFN-γ cytokine could be a negative mechanism intended to suppress the activity of microglial cells in inflammatory conditions. The LAG3 receptor has many ligands, for example, the cell membrane-bound MHC class II proteins, as well as soluble ligands, such as α-synuclein (α-Syn), galectin-3 (Gal-3), and fibrinogen-like protein 1 (FGL1) [113, 114]. It is known that the Gal-3 protein is involved in AD pathology but Gal-3 is a ligand not only for the LAG3 receptor but also for the TREM2 receptor which has been linked with AD pathology [117].

The inhibitory VISTA checkpoint receptor is widely expressed in immune effector cells, especially in T cells and dendritic cells, and also in immunosuppressive cells, such as MDSCs, Tregs, and M2 macrophages [118–120]. The activation of VISTA signaling stimulated macrophage immune tolerance, e.g., lipopolysaccharide (LPS)-induced tolerance and resistance to septic shock in mice, by enhancing the anti-inflammatory properties of macrophages [121]. It is known that the expression of the VISTA protein is suppressed in inflammatory states via the NF-κB signaling pathway [120]. The VISTA receptor has a number of different ligand proteins, such as syndecan-2 (Sdc-2), P-selectin glycoprotein ligand-1 (PSGL-1), and galectin-9 [120]. In the brain, the VISTA receptor is abundantly expressed in human and mouse microglial cells [122, 123]. Borggrewe et al. [122] demonstrated that the expression of the VISTA protein was significantly downregulated in inflammatory disorders, e.g., as induced by LPS exposure and in experimental autoimmune encephalomyelitis (EAE). Borggrewe et al. [123] reported that the expression of the VISTA receptor was significantly reduced in microglial cells isolated from 5xFAD and APP/PS1 mice, probably affected by the inflammatory state. However, the expression of VISTA was elevated in the bulk brain samples obtained from AD mice and post-mortem human AD brains, probably reflecting an infiltration of myeloid cells.

## Involvement of inhibitory checkpoints in AD pathogenesis

### PD-1/PD-L1

Neuroinflammation always involves an interplay between microglia and astroglia in both the aging process and neurodegenerative diseases [124]. Commonly, there are two phenotypes which can be distinguished in both microglial cells and astrocytes, i.e., neurotoxic and neuroprotective phenotypes, which are evident with respect to their transcriptomes and secretomes [124]. In this model, the immunosuppressive pathway involving the PD-1/PD-L1 checkpoint acts as neuroprotective pathway although in certain chronic diseases, such as AD pathogenesis, the activation of PD-1/PD-L1 signaling and its collaboration with immunosuppressive cells might enhance AD pathology. Kummer et al. [53] demonstrated in transgenic AD mice that the expression levels of PD-L1 in astrocytes and PD-1 in microglia were robustly upregulated in the cells surrounding the amyloid deposits. They also observed that astrocytes and microglial cells bordering the amyloid plaques in human AD brains were enriched in the expression of the PD-L1 and PD-1 proteins, respectively. Next, Kummer et al. [53] examined whether a genetic deletion of the microglial PD-1 protein could affect the pathogenesis in the APP/PS1 mice. They reported that the loss of PD-1 in microglia robustly increased the accumulation of amyloid-β (Aβ) within the brains of these transgenic mice. The deprivation of the PD-1 protein significantly reduced the uptake of Aβ into microglia which could be attributed to a decrease in the expression of the microglial CD36 protein, a receptor for Aβ uptake. These results seem to indicate that PD-1/PD-L1 signaling has a crucial role not only in the phagocytosis of Aβ deposits but also in the suppression of neuroinflammation in AD pathology.

**Fig. 2:**
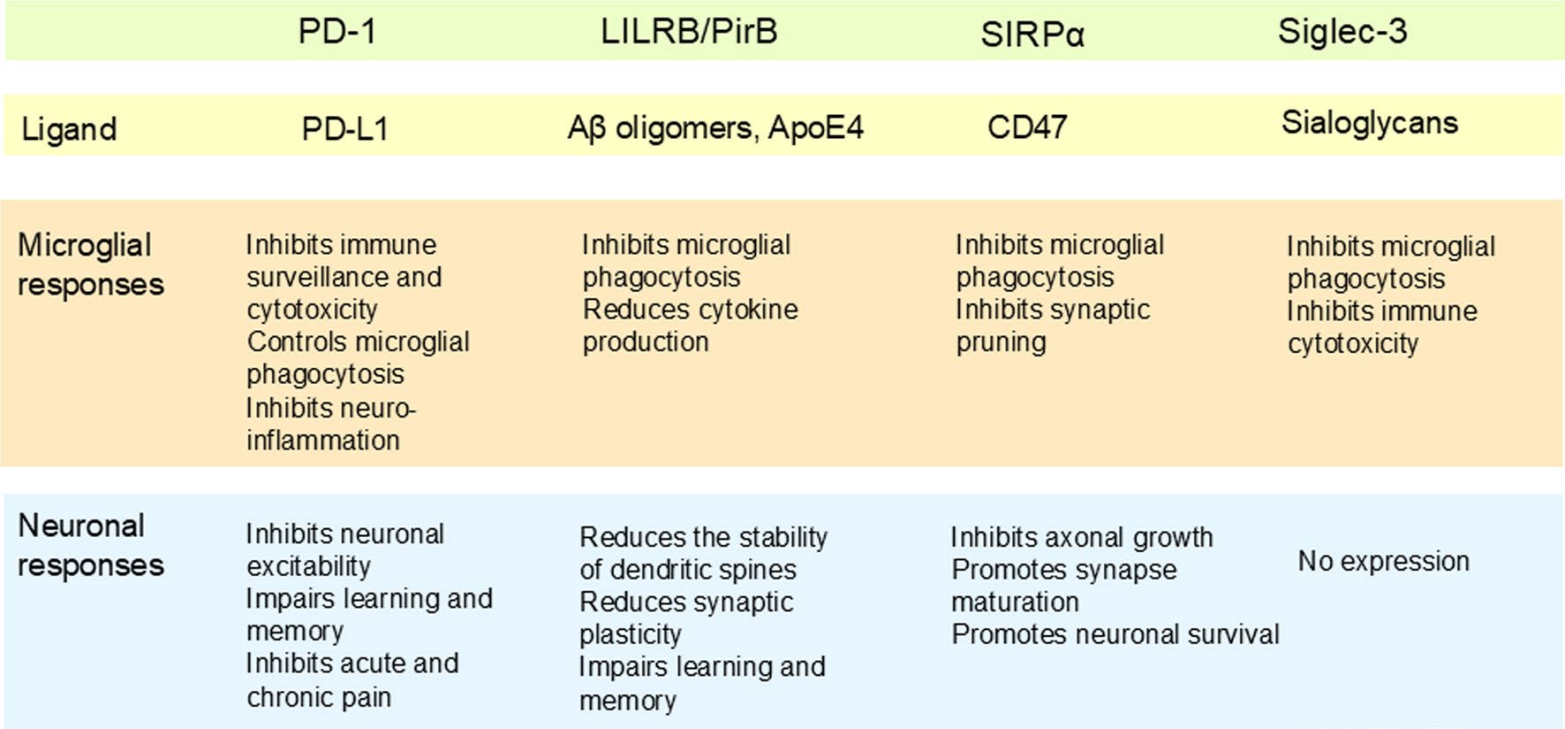
Compilation of microglial and neuronal responses evoked by activation of different inhibitory checkpoint receptors related to the progression of Alzheimer’s disease

However, there are conflicting results on the role of inhibitory PD-1/PD-L1 checkpoint signaling in AD pathogenesis emerging from works done with transgenic mouse models. Baruch et al. [125] demonstrated that a blockade of the inhibitory PD-1 checkpoint receptor by antibody therapy reduced the cerebral accumulation of Aβ plaques in transgenic 5xFAD and APP/PS1 mice. The antibody therapy also improved the cognitive performance of the transgenic AD mice (Fig. 2). They reported that the antibody therapy evoked an IFN-γ-dependent recruitment of monocyte-derived macrophages into the brain that might have reduced the deposition of Aβ peptides in the brains of transgenic AD mice. However, Latta-Mahieu et al. [126] reported that PD-1 immunotherapy with the same antibody as used by Baruch et al. [125] did not alter the cerebral Aβ burden in three other transgenic mouse models of AD. These observations indicated that anti-PD-1 therapy probably did not directly affect the phagocytosis of Aβ plaques. On the other hand, Zou et al. [127] observed that the expression levels of the PD-1 and PD-L1 proteins were robustly upregulated in the cortex and hippocampus of 5xFAD mice. Interestingly, they reported that a blockade of PD-1 with antibody therapy decreased the hyperphosphorylation of tau protein, a common pathological marker detected in AD brains, and also improved the cognitive properties of 5xFAD mice. They demonstrated that the blockade therapy downregulated the activity of GSK3β, a well-known enhancer of tau protein pathology. Moreover, Rosenzweig et al. [128] demonstrated that the treatments with both PD-1 and PD-L1 antibodies displayed similar efficacies in animal models of AD and tauopathy. They reported that the blockade of PD-L1 in 5xFAD mice attenuated the severity of the cerebral pathology, e.g., the plaque burden and gliosis, as well as cognitive decline. In a transgenic tau protein model (double mutant-human TAU protein), targeting PD-L1 protein with antibodies reduced inflammation and deposition of tau protein and the neuronal survival was clearly improved. Rosenzweig et al. [128] also reported that the blockade therapy for PD-1/PD-L1 signaling in tauopathy enhanced the recruitment of monocyte-derived macrophages into the brain. They proposed that a decline in the severity of tauopathy could be linked to the expression of macrophage scavenger receptor 1 (MSR1) in the invading macrophages. It seems that a robust increase in the expression of PD-L1 in astrocytes could be one way to reduce neuroinflammation via the microglial PD-1 receptor and thus alleviate AD pathology.

Some of the conflicting results may be due to the expression of the PD-1 protein in the neurons, especially in the case of the PD-1 immunotherapy (Fig. 2). For instance, Zhao et al. [55] demonstrated that the conditional knockout (KO) of the PD-1 receptor in mouse brain increased neuronal excitability measured as long term potentiation (LTP) in hippocampal neurons. They observed that loss of PD-1 signaling increased the density of dendritic spines in KO neurons. Zhao et al. [55] also reported that suppression of neuronal PD-1 signaling improved the mouse cognitive decline after brain injury. These results suggested that an increase in PD-1/PD-L1 signaling could impair learning and memory, e.g., in AD patients. There are also investigations revealing that neuronal PD-1/PD-L1 signaling inhibited acute and chronic pain via regulating the GABAergic neurotransmission [54]. Chen et al. [54] demonstrated that the PD-1 receptor is expressed in primary sensory neurons of human and mouse dorsal root ganglia (DRG). They revealed that PD-1/PD-L1 signaling suppressed nociceptive neuron activity in mouse DRG. Currently, it is known that the PD-1/PD-L1 pathway can affect different pain models including both immune and neuronal regulation [129].

### Siglecs/sialoglycans

There has been great interest to Siglecs in AD pathogenesis since 2011 when genetic studies revealed that the *CD33/Siclec-3* gene was a risk factor for AD pathology, as reviewed by Estus et al. [130]. Subsequently, Griciuc et al. [131] demonstrated that the expression of the Siglec-3 receptor was robustly increased in the frontal cortex of AD patients. An increased expression of Siglec-3 was localized into microglia in AD patients. They also reported that the expression level of microglial Siglec-3 significantly correlated with the severity of the Aβ pathology in the frontal cortex of AD patients. Moreover, Griciuc et al. [131] demonstrated that an increase in the expression of CD33 protein inhibited the phagocytosis of Aβ deposits by microglial cells (Fig. 2). Interestingly, they also revealed that the SNP rs 3865444, located in the promoter region of the *CD33/Siglec-3* gene, led to a reduction in the expression of the CD33/Siglec-3 protein as well as a protection against AD pathology, e.g., it prevented the deposition of Aβ plaques within the brain. Moreover, deletion of the *CD33/Siglec-3* gene in transgenic APP/PS1 mice significantly reduced the expression level of insoluble Aβ42 and the accumulation of amyloid plaques. Concurrently, Malik et al. [132] revealed that the SNP rs12459419 was coinherited with rs3865444 in the exon 2 in the AD-risk allele. The exon 2 encodes the sialic acid binding domain in the CD33/Siglec-3 receptor protein and thus it is unable to respond to sialylated ligands. Subsequent studies have revealed that the activation of the Siglec-3 receptor inhibited the microglial phagocytosis driven by TREM2 [133, 134]. The TREM2 receptor not only triggers phagocytosis but it is also involved in lipid metabolism and in many immune responses. Currently, it is known that the expression of the Siglec-3 and TREM2 proteins is upregulated by hypoxia/ischemia [135]. Accordingly, there is abundant evidence that the early stages of AD pathogenesis are associated with a hypoperfusion of the vulnerable brain regions leading to hypoxia/ischemia [136–138]. It seems plausible that an activation of the Siglec-3 checkpoint receptor inhibits the microglial-induced phagocytosis thus promoting the accumulation of Aβ plaques within AD brains.

The accumulation of neuritic plaques in AD can be attributable either to an increased generation of Aβ peptides and their aggregation or to deficiencies in their phagocytosis by microglial cells. The activity of microglial phagocytosis can be reduced, e.g., via the activation of the Siglec receptors, or alternatively microglia are not able to recognize Aβ plaques. We have proposed that neuritic plaques could be masked by sialylated “self” markers which inhibit microglial activity [139]. Currently, there is robust evidence that sialylated gangliosides affect the formation and clearance of Aβ plaques [135, 140]. The amount of sialylated gangliosides, such as GM2, GM3, and GD3, is elevated in the brains of AD patients and transgenic AD mice [141, 142]. It is known that sialylated gangliosides, e.g., GM1 and GD1, can bind to Aβ peptides and accumulate within Aβ plaques [143]. Yanagisawa et al. [144] demonstrated that the binding of GM1 to Aβ protein enhanced the formation of Aβ fibrils and aggregates. Several mass spectrometric experiments have demonstrated that neuritic plaques contain sialylated GM1, GM2, and GM3 gangliosides [145, 146]. Sialylated gangliosides were localized to the diffuse periphery of Aβ plaques rather than to the dense Aβ core [145]. Rapoport et al. [147] demonstrated that the CD33/Siglec-3 protein and several CD-33-related Siglecs were able to bind many sialylated gangliosides, such as GM2, GM3, and GD3. Thus, it seems that sialylated gangliosides in neuritic plaques could act as ligands for microglial Siglec receptors and suppress their activity. Bernardo et al. [148] demonstrated that the deletion of the *GD3 synthase* gene in the APP/PS mice clearly reduced the deposition of Aβ plaques and improved the memory of the transgenic AD mice. Accordingly, Dukhinova et al. [149] revealed that the crossing of the 5xFAD mice with mice which lacked the *GM3 synthase* gene (st3gal5), an α2,3-sialyltransferase, generated a phenotype which was deficient in the major brain gangliosides. They reported that these double knockout mice displayed a robust decrease in the level of Aβ deposits and neuroinflammation, probably due to a loss of gangliosides in the Aβ plaques. Given that the α2,3-linkage of sialic acid is a ligand of Siglec-3, this indicates that gangliosides bound to Aβ plaques can inhibit the activity of microglia and suppress the phagocytosis of amyloid plaques. This reveals that not only the glycocalyx of cells but also sialylated plaques can inhibit microglial activity via the inhibitory Siglec checkpoint receptors.

### LILRB2-4/Aβ/ApoE4/fibronectin

There is compelling evidence that the LILRB2/PirB (also called ILT4) receptor is expressed in microglial cells, astrocytes, and some neuronal cells and interestingly, many investigations have revealed that these LILRB2 receptors seem to be involved in the pathogenesis of AD [87, 89, 150–152]. Kim et al. [87] demonstrated that human LILRB2 and mouse ortholog PirB proteins were the receptors for oligomeric Aβ42 peptides with a nanomolar affinity. Recently, the binding mechanism of Aβ42 oligomers to the LILRB2 receptor has been confirmed with molecular docking techniques [151, 153]. Zhao et al. [89] demonstrated that LILRB2 signaling inhibited the function of the TREM2 receptor in human microglial cells. For instance, Aβ42 oligomers were able to inhibit TREM2-mediated microglial phagocytosis. Accordingly, a specific antibody to the LILRB2 receptor blocked not only the signaling triggered by oligomeric Aβ42 but it also promoted phagocytosis by microglia and increased the expression of cytokines in human microglia. They also reported that the blockade of LILRB2 signaling enhanced the microglial phagocytosis of Aβ42 deposits in 5xFAD mice (Fig. 2). Currently, it is possible to exploit the molecular structure-based design techniques to develop inhibitors which can prevent the interaction between Aβ42 oligomers and the LILRB2 receptors in AD brains [151].

In addition to microglia, LILRB2 and its murine orthologue PirB receptor are expressed in the neurons of mouse and human brain [87, 92] (Fig. 2). In 2013, Kim et al. [87] demonstrated that in the brains of transgenic AD mice, the presence of Aβ oligomers impaired hippocampal long-term potentiation, reduced synaptic plasticity, and induced memory deficits in a PirB-dependent manner. They reported that Aβ oligomer-induced loss of spines was mediated by protein phosphatase 2A and 2B-stimulated cofilin activation and actin filament disassembly through the signaling via the PirB receptor. After these seminal observations, Djurisic et al. [93] demonstrated that the conditional deletion of PirB receptors from mouse pyramidal neurons reduced long-term depression (LTD) whereas it significantly increased long-term potentiation (LTP). They reported that the NMDA receptor-mediated synaptic mechanisms maintained synaptic plasticity via activity-dependent endocannabinoid signaling. Subsequently, Albarran et al. [154] demonstrated that the knockout of the PirB receptor increased the stability of newly formed dendritic spines and motor learning was improved in mice by blocking the function of the PirB receptor. Moreover, the knockout of the PirB receptor induced neuroprotective effect on stroke and enhanced brain recovery in mice [155]. Given that loss of synapses and memory deficits are common hallmarks of the early phase of AD pathology, it seems that Aβ-induced signaling via neuronal PirB receptors can promote the pathogenesis of AD.

The inhibitory LILRB3 checkpoint receptor (also called ILT5) has several ligands and it is expressed in many myeloid cells, mostly macrophages and neutrophils but also in microglia [85, 90, 156]. Interestingly, Zhou et al. [90] demonstrated that ApoE4 was a putative ligand for the LILRB3 receptor present on the surface of human microglial cells (HMC3). They reported that the LILRB3 receptor specifically recognized the ApoE4 protein but not the ApoE2 counterpart. They also characterized the structural interaction domains between ApoE4 and the LILRB3 receptors. Zhou et al. [90] demonstrated that ApoE4 treatment of human microglia induced the expression profile of increased interferon, cytokine, and antiviral signaling, whereas LILRB3-ablated cells were unresponsive to ApoE4 treatment. It is known that the ApoE4 isoform significantly increases the risk for late-onset sporadic AD, although the exact mechanism still needs to be clarified.

Recently, Hou et al. [91] revealed that the inhibitory LILRB4 receptor (also termed ILT3) was abundantly expressed in microglia in the brains of AD patients. In transgenic 5xFAD mice, there was a robust expression of LILRB4 protein in the microglia surrounding Aβ plaques. A blockade of LILRB4 signaling reduced the accumulation of Aβ proteins in the brains of 5xFAD mice. This was probably attributed to an improved maintenance of microglial activity, e.g., gene profiling corroborated that anti-LILRB4 exposure improved microglial phagocytosis and inhibited interferon signaling in 5xFAD mice [91]. Accordingly, Kamphuis et al. [157] reported that the expression of LILRB4 was significantly increased in the cortical hemisphere of old transgenic APP/PS1 mice as compared to old wild-type mice. Hou et al. [91] demonstrated that an antibody blockade of LILRB4 prevented the binding of lipidated and non-lipidated mouse ApoE to the LILRB4 receptor. They also applied *in silico* techniques and confirmed that mouse ApoE protein was a putative ligand for the LILRB4 receptor. Although there are several recognized ligands for the LILRB4 checkpoint receptor, nonetheless their role in AD pathogenesis needs to be clarified. For instance, fibronectin is one such ligand, an extracellular matrix protein, which could regulate microglial activity in AD pathology [158]. Lepelletier et al. [159] reported that in the fronto-temporal cortex, the expression of fibronectin was significantly increased in the early phases of AD pathology (Braak stages 2–4). Recently, Bhattarai et al. [158] described a genetic variant rs140926439 in the fibronectin 1 gene (*FN1*) in cognitively unaffected homozygous *APOEε4* carriers (over 70 years) indicating that this variation was protective against AD pathology. They reported that the level of the FN1 protein and reactive gliosis were robustly reduced in the blood–brain-barrier (BBB) of unaffected homozygous *APOEε4* carriers than the *APOEε4* carriers with AD. It could be speculated that the variant FN1 ligand can prevent the activation of LILRB4 signaling induced by other ligands, such as the ApoE protein.

### SIRPα/CD47

Loss of synapses, i.e., synaptic pruning, is a common hallmark encountered in many neurodegenerative diseases, such as in AD [160, 161]. Several investigators have emphasized the role of microglia in the synaptic pruning in AD pathology [162, 163]. As discussed earlier, the function of the SIRPα/CD47 axis is able to control the pruning process in mouse brain [102] (Fig. 2). Interestingly, Ding et al. [102] demonstrated that the level of the SIRPα protein was significantly downregulated in the cortex of AD patients as well as in the brain of transgenic AD mice. They also reported that the expression level of microglial SIRPα decreased in parallel with the increased pathology in transgenic AD mice. Ding et al. [102] also revealed that exposure of mouse primary microglia to the soluble Aβ42 oligomer robustly decreased the expression of the SIRPα protein. Moreover, a specific ablation of the microglial *SIRPα* gene in transgenic APP/PS1 mice accelerated the rate of synaptic loss and the severity of the cognitive impairment in AD mice although it did not increase the accumulation of Aβ plaques. A knockout of the *SIRPα* gene in microglial cells enhanced their phagocytic activity towards synapses, especially upon Aβ stimulation [102]. In in vitro assays, treatment of microglia with Aβ oligomers robustly reduced the expression of the SIRPα protein, whereas it clearly increased the microglial phagocytosis of synaptosomes. Ding et al. [102] also demonstrated that the expression level of the CD47 protein in synaptosomes was significantly reduced in the brain of AD mice. Moreover, the treatment of mouse cultured primary neurons with Aβ oligomers robustly decreased the expression level of the CD47 protein. These observations clearly indicated that disturbances in the SIRPα/CD47 axis have a crucial role in phagocytosis-induced synaptic pruning in AD pathology.

The SIRPα protein is also expressed in neurons where it locates both in the growth cones and synapses (Fig. 2). Wang and Pfenninger [104] demonstrated that the SIRPα protein was localized in the lipid rafts of growth cone periphery in rat cortical neurons. They observed that the exposure of laminin and IGF-1 and BDNF induced phosphorylation of the cytoplasmic tail of SIRPα and increased the binding of SHP-2. The phosphorylation of the cytoplasmic tail inhibited the IGF-1-induced axonal growth on extracellular matrix (ECM). It seems that the SIRPα protein is able to control neurogenesis both in developmental and pathological conditions. Moreover, Nagappan-Chettiar et al. [164] demonstrated that synaptic activation induced phosphorylation of the SIRPα protein on the postsynaptic transmembrane in mouse hippocampal neurons. This phosphorylation stimulated the cleavage of the ectodomain of the SIRPα protein inducing maturation of hippocampal synapses. There are also investigations indicating that the expression of SIRPα can have a significant role in the regulation of neuronal survival [165, 166]. For instance, Araki et al. [165] reported that overexpression of SIRPα/SHPS-1 in cultured rat cortical neurons induced the phosphorylation of the cytoplasmic tail and binding of the SHP-2 protein. The overexpression of the SIRPα protein enhanced BDNF-induced neuronal survival by activating signaling through the Akt/PI-3 K pathway. In the future, the role of the neuronal SIRPα protein in AD pathology needs to be clarified.

Currently, it is known that the membrane-bound CD47 protein can act not only as the ligand to the immune SIRPα receptor but can also function as the receptor for some soluble proteins, such as the matricellular protein thrombospondin-1 (TSP1) [167, 168]. In fact, the affinity of the interaction between the TSP1 and CD47 proteins is higher than the interaction between the SIRPα and CD47 proteins [168]. There is abundant evidence that via CD47 signaling the TSP1 protein can control many cardiac, pulmonary, and vascular responses in both health and disease [169]. For instance, TSP1/CD47 signaling can stimulate senescence of endothelial cells thus promoting vascular diseases [170]. Interestingly, Son et al. [171] demonstrated that the TSP1 protein, secreted by astrocytes, prevented Aβ-mediated synaptic pathology in transgenic AD mice. They reported that the expression levels of TSP1 was robustly decreased in the cortical areas of AD patients and in the hippocampus of AD mice. Son et al. [171] also revealed that Aβ1–42 exposure reduced the expression and release of the TSP1 protein from mouse primary astrocytes and human astroglioma cells. Moreover, Buee et al. [172] observed in AD patients that immunohistochemical staining of the TSP1 protein was significantly downregulated in a subset of pyramidal neurons within the regions vulnerable to AD pathology. All these observations indicate that a decrease in the expression of the TSP1 protein could promote a synaptic loss in AD via the TSP1/CD47 pathway. However, the dynamic regulation between the SIRPα/CD47 and the TSP1/CD47 pathways needs to be revealed.

## Are inhibitory immune checkpoints involved in AD pathogenesis?

As discussed above, there is mounting evidence that signaling via inhibitory immune checkpoint receptors suppresses the function of microglial cells, e.g., the cells lose their phagocytic activity and this promotes the accumulation of Aβ deposits within the vulnerable brain regions. The presence of neuroinflammation increases the expression of ligand proteins which can activate microglial inhibitory checkpoint receptors. For example, it is known that in inflammatory conditions, the NF-κB and JAK/STAT signaling pathways stimulate the transcription of the PD-L1 gene (*CD274*) [173–175]. Moreover, inflammatory and stress-related mediators upregulate the expression of both CD47 protein and fibronectin via the NF-κB signaling pathway [176, 177]. There is substantial evidence that pro-inflammatory senescent cells display a significant upregulation of inhibitory checkpoint ligands, such as PD-L1 [178, 179], fibronectin [180], and CD47 [106]. It should be emphasized that astrocytes display many of the common characteristics of cellular senescence, such as a pro-inflammatory phenotype, not only in the aging process but especially in AD pathology [181–185]. In particular, the astrocytes surrounding amyloid plaques exhibit many of the traits seen in cellular senescence. Emerging studies have revealed that diverse types of senescent cells express an increased level of the PD-L1 protein [178, 179]. An upregulation of the PD-L1 protein prevents their immune surveillance and thus senescent cells can evade immune elimination and subsequently they accumulate within aged and diseased tissues. In mouse brain, Gao et al. [186] demonstrated that a traumatic brain injury (TBI) stimulated a specific increase in the expression of the PD-L1 protein in reactive/senescent astrocytes. They reported that the upregulation of PD-L1 not only counteracted microglia-induced inflammation but also attenuated the severity of the TBI-induced neuronal damage. Recently, Linnerbauer et al. [187] revealed that experimental autoimmune encephalomyelitis in mice significantly increased the expression of PD-L1 in reactive astrocytes and subsequently attenuated inflammatory responses of the PD-1-positive microglia. These results are comparable with those findings discussed above with respect to AD pathology [53, 127] indicating that the astrocytic PD-L1 protein is able to activate microglial PD-1 signaling and thus suppress microglial functions, e.g., impairing their phagocytic activity to remove Aβ deposits.

There is convincing evidence that synaptic dysfunction is an early event in AD pathology and it can even precede the accumulation of Aβ deposits [161, 188]. Synaptic loss is a characteristic feature in the early stage of AD pathology and its level strongly correlates with cognitive decline [188]. Many studies have revealed that soluble forms of Aβ can cause synaptotoxicity associated with calcium dysregulation as well as tau protein and mitochondrial dysfunctions which can lead to learning and memory deficits [188, 189]. Interestingly, there is mounting evidence that neuronal hyperexcitability and synaptic reorganization are associated with early AD pathology in mouse Aβ models [190, 191]. Several mechanisms have been proposed although the Aβ-induced activation of neuronal PirB receptor could provide a perfect model for the regulation of neuronal excitability and synaptic plasticity in the early phase of AD pathology (see above). Given that activation of the PirB receptor impairs the stability of dendritic spines [154], this could explain why dendritic spines are lost in the early phase of AD pathology [192].

The primary cause of late-onset AD is still unknown although its pathology has been extensively investigated and a number of molecular mechanisms postulated. There are many neuroimaging studies indicating that cerebral blood flow is significantly reduced in vulnerable brain regions of AD patients leading to the development of the hypoxic microenvironment [136–138]. For instance, Yang et al. [193] demonstrated that chronic cerebral hypoperfusion accelerated the rate of Aβ pathology in transgenic AD mice. It is known that hypoxia stimulates amyloidogenic processing of amyloid precursor protein (APP) and promotes the accumulation of Aβ deposits [194]. It is not only Aβ deposition which can stimulate the expression of PD-L1 protein [53] but the PD-L1 gene (*CD 274*) is also a direct transcriptional target of hypoxia via an induction of hypoxia-inducible factor 1α (HIF-1α) [195]. For instance, hypoxia is a potent inducer of the expression of PD-L1 in the cancer microenvironment [196] and also in obstructive sleep apnea [197]. Blumenau et al. [198] reported that subacute hypoperfusion induced a robust increase in the expression of Siglec-3/CD33 in the mouse hippocampus after a common carotid artery stenosis. Moreover, hypoxia/ischemia impairs signaling via the Siglec-3/TREM2 pathway in the brain and disturbs the phagocytic activity of microglial cells [135]. It seems plausible that hypoxia could disturb the function of microglial cells in hypoxic brain regions by activating many inhibitory checkpoints and thus promote AD pathogenesis.

There are reports that AD pathology could be induced by viral infections [199–201]. Herpes simplex virus type 1 is a major suspect but there are several other viruses, such as herpes simplex encephalitis, cytomegalovirus, and varicella-zoster virus. Currently, there is some evidence that certain prokaryotic organisms, e.g., spirochetes, chlamydia, and helicobacter pylori, as well as eukaryotic protozoa, such as toxoplasma gondii, could be involved [200, 201]. Interestingly, it has been claimed that Aβ peptide could be an ancient antimicrobial peptide which represents an innate immunity response to combat invasion by pathogens [202]. In this respect, it is interesting that several investigations have indicated that inhibitory immune checkpoints have a crucial role in viral infections [203, 204]. For instance, viral infections impair the function of T cells by activating inhibitory checkpoints, such as PD-1 and LAG3 [204]. The upregulation of inhibitory checkpoint signaling means that infected cells can avoid immune clearance, a similar situation exists in cancer cells. Currently, there are only a few investigations into the mechanisms which could activate the inhibitory checkpoint signaling in viral infections. It is known that viral-infected cells stimulate the expression of the PD-L1 protein which activates the PD-1/PD-L1 signaling axis and subsequently can cause the exhaustion of CD8^+^ T cells [205, 206]. Moreover, viral infections can stimulate the cGAS-STING signaling pathway [207] which is known to induce the expression of PD-L1 in different experimental conditions [208, 209]. Recently, Xie et al. [210] demonstrated that the microglial cGAS-STING pathway was activated in the brains of AD patients. In 5xFAD mice, the deficiency of cGAS in microglia ameliorated Aβ pathology and neuroinflammation. In addition, an inhibitor of the STING protein, H-151, reduced the activation of cGAS-STING signaling and attenuated the severity of AD pathology in 5xFAD mice. It remains to be clarified whether or not these results could be associated with PD-1/PD-L1 signaling.

There is a close cooperation between inhibitory immune checkpoint signaling and the immunosuppressive network. For instance, tumor cells exploit this collaboration between inhibitory checkpoint receptors and immunosuppressive cells, e.g., MDSCs, Tregs, tumor-associated macrophages (TAM), and cancer-associated fibroblasts (CAF) [211]. The cooperation is evident also in many chronic inflammatory diseases, such as many autoimmune diseases [212, 213]. In brief, it is known that PD-1/PD-L1 signaling was able to convert mouse PD-1-positive CD4^+^ T cells into the induced Tregs (iTreg) which consequently suppressed the proliferation and activity of CD4^+^ T cells [47]. There are also many indications that an activation of PD-1/PD-L1 signaling in MDSCs robustly stimulated their proliferation and immunosuppressive functions in tumor microenvironment [214] and also in cases of sepsis [215]. Moreover, Wei et al. [216] demonstrated that treatment of human THP-1 cells with the human recombinant PD-L1 protein induced their polarization toward the immunosuppressive M2 phenotype via PD-1/PD-L1 signaling. On the contrary, MDSCs, Tregs, and M2 macrophages secrete immunosuppressive cytokines, such as TGF-β and IL-10, which stimulate the expression of both the PD-1 and PD-L1 proteins in different experimental conditions [217–219]. Moreover, exposure to TGF-β1 evoked anti-inflammatory responses in microglia [220], and furthermore, an overexpression of TGF-β1 in transgenic AD mice stimulated the accumulation of Aβ deposits in cerebral blood vessels [32]. Currently, the role of infiltrated myeloid cells in AD pathology is largely unknown although there is some evidence that recruited peripheral myeloid cells could affect AD pathogenesis [221, 222]. Interestingly, Reed-Geaghan et al. [223] applied genetic techniques and demonstrated that the Aβ plaque-associated myeloid cells in the AD brain were derived exclusively from resident microglia and not from the circulating myeloid pool. Moreover, Keren-Shaul et al. [17] revealed that the plaque-associated, highly phagocytic microglial cells were converted from their homeostatic state to a disease-associated microglial (DAM) phenotype. They also reported that the activation of this phagocytic state was inhibited by microglial inhibitory checkpoint pathways. Given that the DAM cells are unable to remove Aβ plaques, it seems plausible that an activation of inhibitory checkpoint signaling is involved in the suppression of microglial phagocytosis and thus promoting AD pathogenesis.

## Conclusions

Currently it is known that microglial cells have a crucial role in the pathogenesis of AD. The immune hyporesponsiveness of microglia to remove Aβ deposits promotes a chronic neuroinflammation, induces a loss of synapses, and impairs cognitive activities. A decline in the immune surveillance properties of microglia enhances the accumulation not only of senescent cells but also pathogens within the AD brain. The cause for microglial immune deficiency in AD pathology still needs to be clarified. However, it is known that the activation of the inhibitory checkpoint receptors present in immune cells impairs their capacity to combat tumor growth and respond to chronic pathogen insults. Microglial cells possess several inhibitory checkpoint receptors, such as PD-1, LILRB2-4, Siglec, and SIRPα receptors. Moreover, many neuronal cells in the brain also contain inhibitory checkpoint receptors although their functions still needs to be clarified. Currently, it seems that they are involved in the regulation of synaptic structures, such as density of dendritic spines, as well as learning and memory properties. These functions suggest that the activation of neuronal checkpoint receptors could have a role in the early phase of AD pathology. Interestingly, the microenvironment of the AD brain contains an increased amount of ligands for inhibitory checkpoint receptors which are able to suppress the function of the microglia. For instance, an accumulation of Aβ42 oligomers, ApoE4, and fibronectin can stimulate the microglial LILRB2-4 receptors. Similarly, an elevated amount of sialic acid containing gangliosides inhibits the Siglec receptors of microglia. Moreover, AD pathology involves an excessive level of senescent cells which typically express a high level of the PD-L1 proteins which in turn can stimulate the PD-1 receptors of microglia and impair their immune surveillance activity. Instead, a decrease in the function of the SIRPα/CD47 pathway in AD brain stimulates the phagocytic pruning of synapses in neurons as occurs in the pathogenesis of AD. In summary, there are many different sources for neuroinflammation and consequently a plethora of ways that can lead to disturbances in the signaling of the inhibitory checkpoints of microglia in the AD brain. For instance, an increase in amyloidogenic processing of APP generates Aβ deposits which might be partially induced by hypoxic insults in vulnerable brain regions. Viral infections could also enhance the deposition of Aβ peptides in AD and impair the immune surveillance properties of microglia as has been detected in viral infections in other tissues. In conclusion, it seems plausible that inhibitory immune checkpoint signaling has a crucial role in the pathogenesis of AD.

## Data Availability

Not applicable.
